# Memory-driven attentional capture reveals the waxing and waning of working memory activation due to dual-task interference

**DOI:** 10.3758/s13423-016-1041-6

**Published:** 2016-04-28

**Authors:** Edyta Sasin, Mark Nieuwenstein

**Affiliations:** Department of Experimental Psychology, University of Groningen, Grote Kruisstraat 2/1, 9712 TS Groningen, The Netherlands

**Keywords:** Working memory, Attentional capture, Memory-driven capture

## Abstract

Previous studies have shown that information held in working memory (WM) actively or as a residue of previous processing can lead to attentional capture by corresponding stimuli in the environment. Here, we compared attentional capture by goal-driven and residual WM activation and examined how these effects are affected by dual-task interference. In two experiments, participants performed an animacy judgment task for a word that they did or did not have to remember for a later recognition test. The word was followed in half of the trials by an arithmetic task that served to disrupt the WM activation of the previously processed word. Subsequently, WM-driven capture was assessed by having participants perform a single-target rapid serial visual presentation task in which a line drawing corresponding to the word was presented shortly before a target. The results showed that the line drawing captured attention irrespective of the presence of the arithmetic task when the word had to be remembered. In comparison, the animacy judgment alone resulted in capture only when the arithmetic task was absent, and this effect was equally strong as the capture effect caused by a to-be-remembered word. Taken together, these findings show that although residual and goal-driven WM activation may be equally potent in guiding attentional selection, these two forms of WM activation differ in that residual activation is overwritten by an attention-demanding task, whereas goal-driven WM activation can lead to the reinstatement of a stimulus after performing such a task.

Working memory (WM) enables the short-term maintenance and manipulation of goal-relevant information (Baddeley, 1992; Cowan, 2005). Since the capacity of WM is limited (e.g., Cowan, 2010; Luck & Vogel, 1997), there is a need for the prioritization of relevant over irrelevant information, so as to ensure that only relevant information is represented in WM. This prioritization is assumed to be driven by a target template that is held in WM, and that biases attention to matching items in the visual field (Desimone & Duncan, 1995). In support of this idea, many studies have shown that holding a stimulus in WM can lead to attentional capture by a matching stimulus even when this stimulus is irrelevant for a current task (Downing, 2000; Olivers, Peters, Houtkamp, & Roelfsema, 2011; Soto, Hodsoll, Rothstein, & Humphreys, 2008). For example, Soto and colleagues (Soto, Heinke, Humphreys, & Blanco, 2005; Soto, Humphreys, & Heinke, 2006) found that participants were slower to find a target in a search task if one of the distractors matched a colored shape that was being held in memory for a later memory test.

Interestingly, the occurrence of memory-driven capture does not appear to require the active maintenance of information in WM. Instead, it has been found that verbalizing (Soto & Humphreys, 2007), imagining (Pashler & Shiu, 1999), or judging the semantic properties of (Sasin, Nieuwenstein, & Johnson, 2015) a stimulus can also lead to capture by a matching stimulus that is subsequently presented as part of another task. Under these conditions, the processing of the stimulus appears to result in a residual form of WM activation that continues to influence the selection of new information until this activation has dissipated due to decay or interference. In support of this idea, Sasin and colleagues (2015) found that performing an animacy judgment for a word led to capture by a picture matching that word in a subsequent rapid serial visual presentation (RSVP) task, and they found that this effect did not occur when the animacy judgment task and the subsequent RSVP task were separated by the appearance of a display of to-be-remembered visual stimuli. This finding was interpreted as evidence that the activation of the word in WM was overwritten by the information that had to be encoded for the visual WM task, thus preventing attentional capture by a matching picture in the subsequent RSVP task.

## The present study

In view of previous findings demonstrating that both goal-driven and residual WM activation can lead to memory-driven attentional capture, an interesting question concerns whether and how these two forms of WM activation differ in their abilities to guide the selection of new information from the environment. To address this matter, we aimed to replicate our earlier findings that capture driven by residual WM activation from an animacy judgment task can be prevented by means of an intervening task (Sasin et al., 2015), and we aimed to expand upon this work by investigating how the requirement to also remember the target for the animacy task—thus turning the residual activation into goal-driven WM activation—would affect attentional capture under conditions with and without an intervening task.

In considering the potential differences between the capture effects driven by goal-driven and residual WM, it is important to note that the likelihood of memory-driven capture has been argued to depend on the degree of activation of an item held in WM, such that items that are activated more strongly are more likely to result in attentional capture (Olivers et al., 2011). By implication, the comparison of attentional capture caused by residual and goal-driven WM activation can shed light on whether the instruction to remember leads to stronger WM activation than that resulting from merely performing an animacy judgment task, in which case WM might be expected to be weaker due to temporal decay (e.g., Barrouillet, Bernardin, & Camos, 2004).

Although the comparison of attentional capture in the absence of dual-task interference may thus shed light on the temporal decay of residual WM activation, another interesting question is whether the presence of an intervening task would reduce attentional capture when the target for the animacy judgment task had to be remembered. On the one hand, one could argue that the intervening task would be expected to interfere with WM activation for the target word, thereby reducing the likelihood of attentional capture in the subsequent RSVP task. At the same time, however, it could be that the requirement to remember the word would lead to reactivation of the word’s representation in WM after executing the intervening task, thus countering the interference produced by this task by reinstating the representation of the word in WM prior to the RSVP task (Oberauer, Lewandowsky, Farrell, Jarrold, & Greaves, 2011; see also Unsworth & Engle, 2006, 2007).

## Method

### Experiment 1a

#### Participants

Sixty-seven students (43 females, 24 males; *M* = 20 years, *SD* = 2.24) of the English-language psychology bachelor program at the University of Groningen participated in the experiment for partial course credit. All had normal or corrected-to-normal visual acuity. The study was approved by the Ethics Committee of the Psychology Department. Informed written consent was obtained.

#### Apparatus and stimuli

Stimulus presentation and response collection were controlled by a program written with E-Prime 2.0 (Schneider, Eschmann, & Zuccolotto, 2002), and the experiment was done on computers that were fitted with 22-in. CRT monitors with a refresh rate of 100 Hz and a resolution of 1,024 × 768 pixels.

The word stimuli used in the experiment were 64 high-frequency English nouns of high imageability [479–655 (*M* = 593.88) according to the Paivio, Yuille, & Madigan, 1968, norms]. The English Lexicon Project database (Balota et al., 2007) was used to select words of high frequency according to the Hyperspace Analogue to Language (HAL) frequency norms (Lund & Burgess, 1996). We selected words that had a frequency of 20 per million or greater (Brysbaert & New, 2009) and that had been found to be familiar to the participants in our previous study (Sasin et al., 2015). All words were displayed in Courier New, 25-point font. The picture stimuli were line drawings taken from the Snodgrass and Vanderwart (1980) and International Picture Naming Project (Szekely et al., 2005) sets. Drawings of 178 nouns were used as the stimuli in the experiment (64 were used as targets or as fillers in other trials, and 114 were used as fillers), and each measured approximately 10.2 × 10.2 cm (7.29° of visual angle). The arithmetic problems were drawn from a pool of problems that included all possible multiplications of single digits from 2 to 9. Each arithmetic problem consisted of the presentation of a multiplication of two digits and an answer that either was correct or was incorrect by a difference of 2 (e.g., 5 × 6 = 28?). An additional set of 12 words and 40 pictures were selected for a practice block. All of the stimuli were displayed in black on a white background at the center of the screen.

#### Procedure

**Fig. 1:**
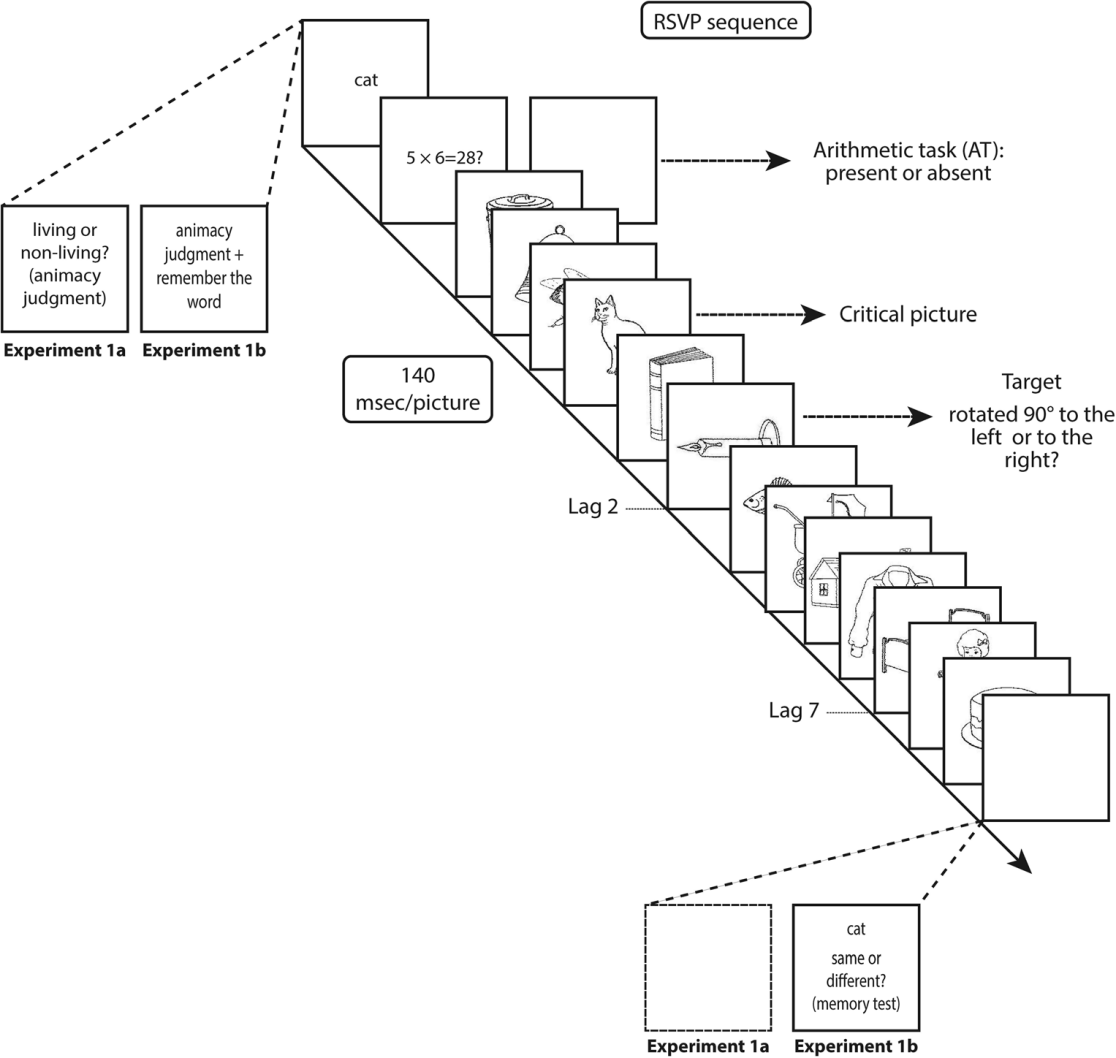
Illustration of the trial sequences in Experiments 1a and 1b

As is shown in Fig. 1, each trial began with the presentation of a word, and participants had to judge whether the word referred to a living or a nonliving thing as quickly as possible, by pressing the “Z” or “M” key of the keyboard. After this animacy judgment task, an arithmetic problem was presented in half of the trials. In this arithmetic-task-present (*AT-present*) condition, participants had to solve the arithmetic problem as quickly as possible by indicating whether the presented answer for the problem was true or false, again using the “Z” and “M” keys. In the condition without the arithmetic task (i.e., the *AT-absent* condition), a blank screen was presented in lieu of the arithmetic problem for a duration of 1,500 ms. Following this blank interval, or following the response to the problem in the AT-present condition, there was a 500-ms fixation period, after which an RSVP stream of 13 pictures (each presented for 140 ms) was displayed. The participant’s task was to search for a target picture that was rotated 90° to the left or the right. A picture corresponding to the target for the animacy judgment task was always presented in Position 4 of the RSVP sequence, and this *critical* picture was followed at a lag of 2 or 7 by the target picture, meaning that the target could appear in the second or the seventh RSVP position following the critical picture. After the sequence had finished, participants responded by pressing the “Z” key for a target picture rotated to the left, and the “M” key for a target picture rotated to the right. Participants were instructed to execute this task as accurately as possible, without time pressure. The experiment consisted of 64 trials, and it was preceded by a 12-trial practice phase. The entire experiment lasted approximately 30 min.

### Experiment 1b

#### Participants

Seventy-nine students (49 females, 30 males; *M* = 20.1 years, *SD* = 1.57) of the English-language psychology bachelor program at the University of Groningen participated in the experiment for partial course credit. All had normal or corrected-to-normal visual acuity. The study was approved by the Ethics Committee of the Psychology Department. Informed written consent was obtained.

#### Apparatus and stimuli

The apparatus and stimuli were identical to those of Experiment 1a, except that additional words were selected for a memory test. We selected 32 nouns of high imageability, 479–655 (*M* = 593.88) according to the Paivio et al. (1968) norms, and high frequency according to the HAL frequency norms (Lund & Burgess, 1996). Half of the words denoted living things, and the other half denoted nonliving things. An additional six words were selected for a practice phase.

#### Procedure

The practice phase and experimental task were the same as in Experiment 1a, except that here participants were asked to remember the word that had been used in the animacy judgment task for a later recognition test. This recognition test was done after participants had responded to the RSVP task, and it required participants to indicate whether a newly presented word was the same as the word they had been asked to remember. The words used in the recognition test were selected at random from the set of available words, and they always had the same animacy category as the word used in the animacy judgment task.

#### Data analysis

We conducted conventional null-hypothesis significance tests, supplemented by Bayes factor analyses to ascertain evidence in favor of a null effect in case the significance test produced a nonsignificant effect. In computing Bayes factors, we used the JASP software package (Love et al., 2015) to compute the evidence for a null effect, with Bayes factors greater than 1 signifying evidence in favor of the null.

## Results

### Experiment 1a

**Fig. 2:**
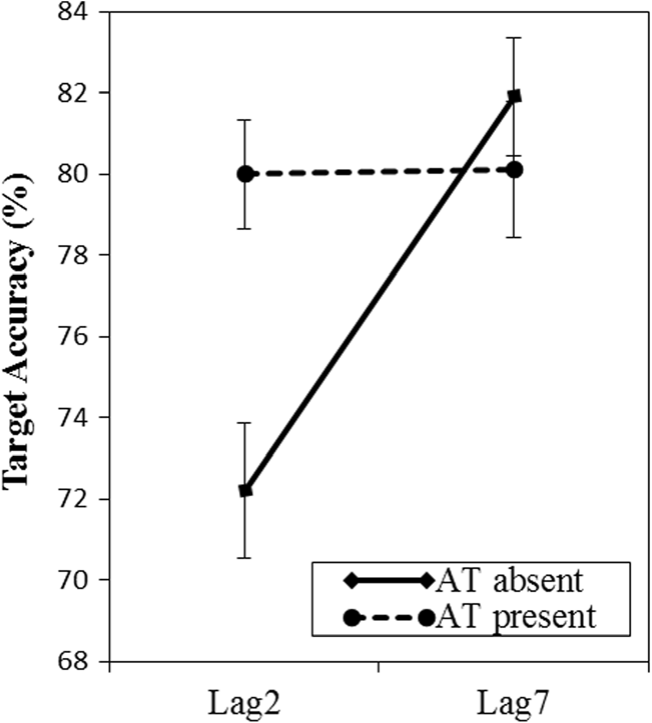
Results of Experiment 1a. Mean accuracy in the RSVP task is plotted as a function of presence of the arithmetic task and lag. The target picture appeared at either lag 2 or lag 7 after the *critical* picture, which depicted the word that had previously been processed for the animacy judgment task. Error bars reflect standard errors of the means

First, we excluded nine participants whose accuracy in the picture identification task was at chance level, as established by a one-tailed binomial test. Exclusion of these participants did not change the pattern of results. Animacy judgments were correct on 97 % of the trials, and the mean RT was 1,308 ms. Performance in the arithmetic task was 94 % correct, and the mean RT was 2,257 ms. The analysis of performance in the RSVP task was restricted to trials that included correct responses in both the animacy judgment task and the arithmetic task—if present. A two-way repeated measures analysis of variance (ANOVA) with the factors Presence of the Arithmetic Task (present vs. absent) and Lag (2 or 7) was performed on the mean accuracies in the RSVP task. The ANOVA revealed no main effect of the presence of the arithmetic task, *F*(1, 57) = 1.23, *p* = .272, Bayes factor = 2.04; a significant effect of lag, *F*(1, 57) = 6.78, *p* = .012, partial *η* ^2^ = 11; and a significant interaction of presence of the arithmetic task and lag, *F*(1, 57) = 4.30, *p* = .043, partial  *η* ^2^ = .07. A follow-up pairwise *t* test confirmed what can be seen in Fig. 2, namely that accuracy was significantly worse at lag 2 (75.2 %) than at lag 7 (81.9 %) when the arithmetic task was absent, *t*(57) = 3.22, *p* = .002, *d* = 0.42, but not when the arithmetic task was present (80 % vs. 80.1 %), *t*(57) = 0.042, *p* = .967, Bayes factor = 6.96.

Taken together, the results of Experiment 1a replicate our earlier findings (Sasin et al., 2015) that an animacy judgment task for a word results in attentional capture by a picture depicting this word in a subsequent RSVP task. As a result of this attentional capture effect, an attentional blink occurred for discrimination of the subsequent RSVP target. Furthermore, the results of Experiment 1a also replicated our earlier finding that the capture effect caused by residual WM activation is abolished by the requirement to perform an attentionally demanding task after the word-judgment task, a finding signified by a Bayes factor of 6.96 in favor of the hypothesis that there was no difference in performance between target discrimination performance at lags 2 and 7. What remained to be determined in Experiment 1b was whether this attentional capture effect would be more pronounced and be unaffected by the arithmetic task if participants were instructed to remember the target word for the animacy judgment task.

### Experiment 1b

**Fig. 3:**
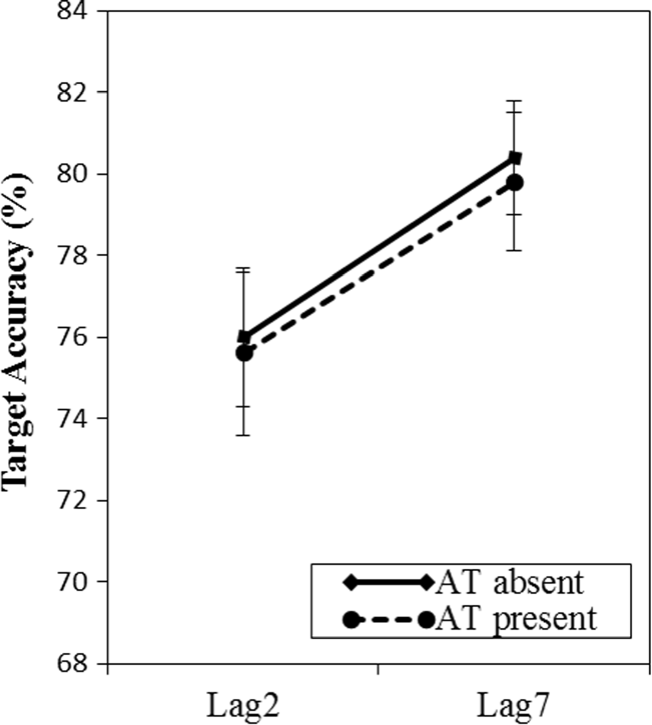
Results of Experiment 1b. Mean accuracy in the RSVP task is plotted as a function of presence of the arithmetic task and position of the target picture. The target picture appeared at either lag 2 or lag 7 after the *critical* picture. Error bars reflect standard errors of the means

The results of 16 participants were excluded from the analysis because of chance-level performance in the picture identification task. Importantly, however, exclusion of these participants from the analysis did not change the pattern of results. The mean accuracy in the animacy judgment task was 97 %, and the mean RT was 1,402 ms. Performance in the arithmetic task was 95 % correct, whereas performance in the memory task was 93 % correct, with mean RTs of 2,437 and 1,329 ms, respectively. We restricted the analysis of performance in the RSVP task to trials with correct responses in the animacy judgment task, the arithmetic task, and the memory task. The results of this analysis showed no main effect of the presence of the arithmetic task, *F*(1, 62) = 0.12, *p* = .730, but a significant effect of lag, *F*(1, 62) = 9.71, *p* = .003, partial *η* ^2^ = .14, with worse accuracy at lag 2 (75.8 %) than at lag 7 (80.1 %). The effect of the arithmetic task did not interact with the lag, *F*(1, 62) = 0.003, *p* = .958, Bayes factor = 7.23, indicating that the requirement to remember the word led to attentional capture, regardless of the presence of the intermediate arithmetic task (see Fig. 3).

### Results of a comparison of Experiments 1a and 1b

To determine whether the requirement to remember the word in Experiment 1b indeed led to a different pattern of results from those observed in Experiment 1a, we conducted an additional analysis to compare the capture effects observed in these experiments. To this end, we first computed an attentional capture score by subtracting the accuracy score at lag 2 from the score at lag 7, thus producing an estimate of the magnitude of the attentional blink produced by the critical picture. Then we carried out a mixed ANOVA using Experiment as a between-subjects factor and Presence of the Arithmetic Task as a within-subjects factor. Even though the results of Experiments 1a and 1b clearly showed different effects of the presence of the arithmetic task within the two experiments, comparison of the results between experiments showed that this difference did not result in a significant interaction between experiment and the effect of the arithmetic task, *F*(1, 119) = 1.95, *p* = .166, with a Bayes factor of 0.58 signifying that the evidence was inconclusive with regard to the presence or absence of an interaction. Importantly, however, a planned comparison of the magnitudes of the attentional capture effects without the arithmetic task did suggest that there was no difference in attentional capture under conditions in which the target for the animacy judgment task did or did not have to be remembered, *t*(119) = 0.83, *p* = .41, Bayes factor = 3.8, highlighting the fact that, in the absence of the arithmetic task, residual and goal-driven WM activation produced equally strong capture effects in the RSVP task.^1^

## Discussion

In the present study, we compared the extents to which goal-driven and residual WM activation result in attentional capture by a matching stimulus, and we examined how these effects are affected by dual-task interference. Replicating the results of our earlier study (Sasin et al., 2015), the results of Experiment 1a showed that an animacy judgment task for a word results in attentional capture by a picture of that word when it is subsequently shown as a distractor just before a target in RSVP, and they showed that this effect does not occur when participants are asked to first perform an arithmetic task after the animacy judgment task. In contrast, the results of Experiment 1b showed that when participants received the additional instruction to remember the target for the animacy judgment task, attentional capture occurred regardless of the presence of the arithmetic task. Finally, the results of a between-experiment comparison showed that for a condition without the intervening arithmetic task, the attentional capture effects were equally strong, regardless of whether participants were instructed to remember the target for the animacy judgment task.

In considering the implications of the present findings, a first point of discussion lies in the mechanism by which performing an animacy judgment task leads to attentional capture when there is no need to remember the target for this task, as we demonstrated in Experiment 1a. One question that may arise in considering the nature of this mechanism is whether it should be explained in terms of capture driven by residual WM activation, or whether it may be better explained in terms of conceptual priming—that is, a facilitation of processing of the critical picture in RSVP due to the previous processing of the corresponding word in the animacy judgment task. There are several arguments against the latter, priming account. To start with, a key argument derives from the finding that the presence of an intervening arithmetic task prevented the occurrence of attentional capture in Experiment 1a. This finding opposes a priming account because conceptual priming effects are known to persist across intervals of seconds, minutes, and sometimes even days filled with intervening tasks (Woltz & Was, 2007). Another argument against a priming account can be found in a study by Davenport and Potter (2005). In this study, a target word in RSVP could be primed by a related word shown before the RSVP sequence, and the results showed that this semantic priming manipulation did not influence the extent to which the target in RSVP attracted attention. Finally, Soto and colleagues (2005) examined whether the mere presentation of a visual shape leads that shape to subsequently capture attention in a visual search task. The results showed that this form of repetition priming did not lead to attentional capture. Accordingly, we can conclude that the capture effect elicited by a previously processed word in Experiment 1a is unlikely to have been due to priming, because such a priming effect would not be expected to result in attentional capture, and it also would not be expected to be abolished by an intervening task.

In demonstrating that performing an animacy judgment task leads to comparable attentional capture effects regardless of whether the target for this task needs to be remembered, the present findings resonate well with those reported by Soto and Humphreys (2007), who also found comparable attentional capture effects when a visual stimulus had to be verbalized or encoded into memory. Given that the likelihood of attentional capture is assumed to depend on the level of WM activation achieved by an item (Olivers et al., 2011), this set of findings suggests that the level of WM activation that results from an animacy judgment or a verbalization task is comparable to the level of activation that results from asking participants to remember a stimulus. An important conclusion that can be derived from these findings is that information that is activated in WM in the process of executing a certain task is not lost rapidly due to temporal decay if there is no goal to maintain that information for a later memory test, because such a rapid decay of activation should result in a weaker attentional capture effect for residual than for goal-driven WM activation. By implication, the present findings also suggest that it may be unnecessary to include a memory task to study memory-driven attentional capture, because the residual activation that results from processing a stimulus appears to suffice to elicit this effect.

Although residual WM activation may thus be equally as potent as goal-driven WM activation in guiding the focus of attention toward matching stimuli in the environment, it does appear that goal-driven activation is different, in that it allows for capture to occur even after performing an unrelated, attention-demanding task. In this regard, the present findings differ from those of previous studies in which the inclusion of an additional task—the requirement to perform articulatory suppression throughout the trial sequence—was found to result in a lack of attentional capture (Soto & Humphreys, 2008; Woodman & Luck, 2007). In explaining why the present manipulation of including an arithmetic task did not similarly reduce the likelihood of attentional capture, an important consideration lies in the fact that whereas articulatory suppression imposes an increased demand on processing resources throughout the trial sequence, the present study involved only an intermittent increase in load because the arithmetic task was inserted between the encoding of the to-be-remembered item and the subsequent RSVP task. By implication, the present finding that the presence of the arithmetic task did not attenuate attentional capture in the subsequent RSVP task may be explained by assuming that whereas the arithmetic task temporarily displaced the to-be-remembered word from the focus of attention in WM, the instruction to remember the word not only allowed it to be retained in WM during the execution of the arithmetic task, but also allowed for the memory trace to be refreshed after executing the arithmetic task. This account converges with theories of performance in the operation-span task that assume that the execution of an arithmetic task causes the momentary displacement of a to-be-remembered item from the focus of attention to a secondary form of WM, from which it is subsequently retrieved automatically so as to be reinstated in WM after processing of the arithmetic task (Oberauer et al., 2011; see also McCabe, 2008; Unsworth & Engle, 2007).

Taken together, the present findings add to a growing body of research that illustrates how memory can influence the perception of newly encountered information by guiding the focus of attention toward information that matches information activated in the mind (Olivers et al., 2011; Soto et al., 2008). What this work has shown is that when there is no need to maintain a search template actively in WM because it remains unchanged across trials, as in the present experiments, the activation of information in WM appears to be capable of such guidance even when this is detrimental to performing the task at hand, and even when the match between the contents of WM and the incoming sensory input is conceptual in nature. In contrast, when the search template changes from trial to trial, it appears that the template needs to be activated more strongly (Olivers, 2009), thus preventing attentional capture for other items that are concurrently represented in WM (Downing & Dodds, 2004; Houtkamp & Roelfsema, 2006). Importantly, however, the degree of WM activation for a particular item may vary dynamically over time (see also Greene, Kennedy, & Soto, 2015), thus modulating the likelihood of attentional capture, as was also indicated by the present finding that attentional capture varied with the waxing and waning of WM activation caused by momentary distraction.

## Footnotes

1 A comparable analysis of the capture effect in the condition with an arithmetic task showed that the difference between experiments was nonsignificant, *t*(119) = 1.3, *p* = .19, with a Bayes factor of 0.41. Thus, even though the within-subjects comparisons of attentional capture for each experiment separately clearly showed different patterns of results for the individual experiments, the between-experiment comparison was not sufficiently powerful to yield a statistically significant difference for the condition in which the arithmetic task was present.
